# Identification of potential biomarkers of gout through weighted gene correlation network analysis

**DOI:** 10.3389/fimmu.2024.1367019

**Published:** 2024-04-15

**Authors:** Xinyi Wang, Bing Yang, Tian Xiong, Yu Qiu, Yingfen Qin, Xinghuan Liang, Decheng Lu, Xi Yang

**Affiliations:** ^1^ Department of Endocrinology, First Affiliated Hospital, Guangxi Medical University, Nanning, China; ^2^ Department of Geriatric Endocrinology and Metabolism, First Affiliated Hospital, Guangxi Medical University, Nanning, China; ^3^ Department of Endocrinology, Wuming Hospital, Guangxi Medical University, Nanning, China; ^4^ Guangxi Key Laboratory of Precision Medicine in Cardio-cerebrovascular Diseases Control and Prevention, Nanning, China; ^5^ Guangxi Clinical Research Center for Cardio-cerebrovascular Diseases, Nanning, China

**Keywords:** gout, CXCL8, CXCL2, CXCL1, WGCNA

## Abstract

**Background:**

Although hyperuricemia is not always associated with acute gouty arthritis, uric acid is a significant risk factor for gout. Therefore, we investigated the specific mechanism of uric acid activity.

**Methods:**

Using the gout-associated transcriptome dataset GSE160170, we conducted differential expression analysis to identify differentially expressed genes (DEGs). Moreover, we discovered highly linked gene modules using weighted gene coexpression network analysis (WGCNA) and evaluated their intersection. Subsequently, we screened for relevant biomarkers using the cytoHubba and Mcode algorithms in the STRING database, investigated their connection to immune cells and constructed a competitive endogenous RNA (ceRNA) network to identify upstream miRNAs and lncRNAs. We also collected PBMCs from acute gouty arthritis patients and healthy individuals and constructed a THP-1 cell gout inflammatory model, RT−qPCR and western blotting (WB) were used to detect the expression of C-X-C motif ligand 8 (CXCL8), C-X-C motif ligand 2 (CXCL2), and C-X-C motif ligand 1 (CXCL1). Finally, we predicted relevant drug targets through hub genes, hoping to find better treatments.

**Results:**

According to differential expression analysis, there were 76 upregulated and 28 downregulated mRNAs in GSE160170. Additionally, WGCNA showed that the turquoise module was most strongly correlated with primary gout; 86 hub genes were eventually obtained upon intersection. IL1β, IL6, CXCL8, CXCL1, and CXCL2 are the principal hub genes of the protein–protein interaction (PPI) network. Using RT−qPCR and WB, we found that there were significant differences in the expression levels of CXCL8, CXCL1, and CXCL2 between the gouty group and the healthy group, and we also predicted 10 chemicals related to these proteins.

**Conclusion:**

In this study, we screened and validated essential genes using a variety of bioinformatics tools to generate novel ideas for the diagnosis and treatment of gout.

## 1 Introduction

Gout is a common type of arthritis that heals on its own. The prevalence of gout varies greatly between races and geographical areas, with rates ranging from less than 1% to 6.8% (1). The latest projections indicate that gout mortality may increase by 55% in 2060 (2), and the frequency and incidence of this disease have increased globally (3–5). Monosodium urate crystals accumulate in joints, tendons, and other tissues because of persistently high serum urate levels, which causes gout and recurrent episodes of severe, acute, painful arthritis. Gout is more common in older people than in younger people and is related to more risk factors, such as obesity, chronic kidney disease (CKD), and cardiovascular disease (CVD), all of which have a negative effect on patients (6, 7). There are four phases of gout: persistent hyperuricemia, acute inflammation generated by monosodium urate (MSU) crystal activation of immune cells, deposition of MSU crystals, and chronic gouty bone deterioration (8, 9).

MSU is an injury-related molecule whose accumulation in the joint does not always result in inflammation. Initially, MSU stimulates NF-κB via TLR4 and TLR2 and synthesizes pro-IL-1β and NLRP3. Second, MSU crystals also lead to the assembly of NLRP3 and the activation of caspase-1, the proteolytic hydrolysis of which results in the maturation of IL-1β. Ultimately, neutrophils and other cells are transported to the site of crystal deposition, and innate stimulatory immune pathways are activated when IL-1β interacts with IL-1β-interacting receptors, initiating a downstream signaling cascade that includes proinflammatory cytokines and chemokines (9, 10). A reduction in joint inflammation is an essential component of gout treatment. Oral prednisolone, nonsteroidal anti-inflammatory medications, and low-dose colchicine are the most used clinical treatments for gout flare control. However, these oral medicines have diverse modes of action and can cause side effects (10–13). As a result, biomarkers capable of guiding the prevention and treatment of acute gouty arthritis, as well as specific medications, are urgently needed.

Bioinformatics, which includes genomics, proteomics, gene regulation, biological networks, and other fields, employs computer technology to analyze and process enormous amounts of biological data to help researchers obtain a better understanding of disease mechanisms. Weighted gene coexpression network analysis (WGCNA) is a widely used bioinformatics method (14, 15) that clusters highly correlated genes into the same module to reveal functional associations between genes, reflect the biological processes or functions in which these genes are involved, and assess the correlations between gene features and clinical characteristics to discover potential biomarkers or new therapeutic targets. The AGA transcriptome dataset GSE160170 was used in our study to screen for differentially expressed genes (DEGs), and WGCNA was used to identify the most relevant modules for gout. Next, we screened for genes that overlapped between DEGs and the most relevant modules, analyzed these overlapping genes using GO and KEGG functional enrichment analyses, and constructed a protein−protein interaction (PPI) network. Finally, we identified hub genes and validated them using population blood samples and a THP-1 cell model. Using these hub genes, we investigated the connections between immune cells, generated competing endogenous RNA (ceRNA) networks, and predicted target drugs. Through the identification of biomarkers indicative of acute gouty arthritis diagnosis and treatment, we hope to identify new therapeutic options for this disease.

## 2 Materials and methods

### 2.1 Dataset collection

**Figure 1:**
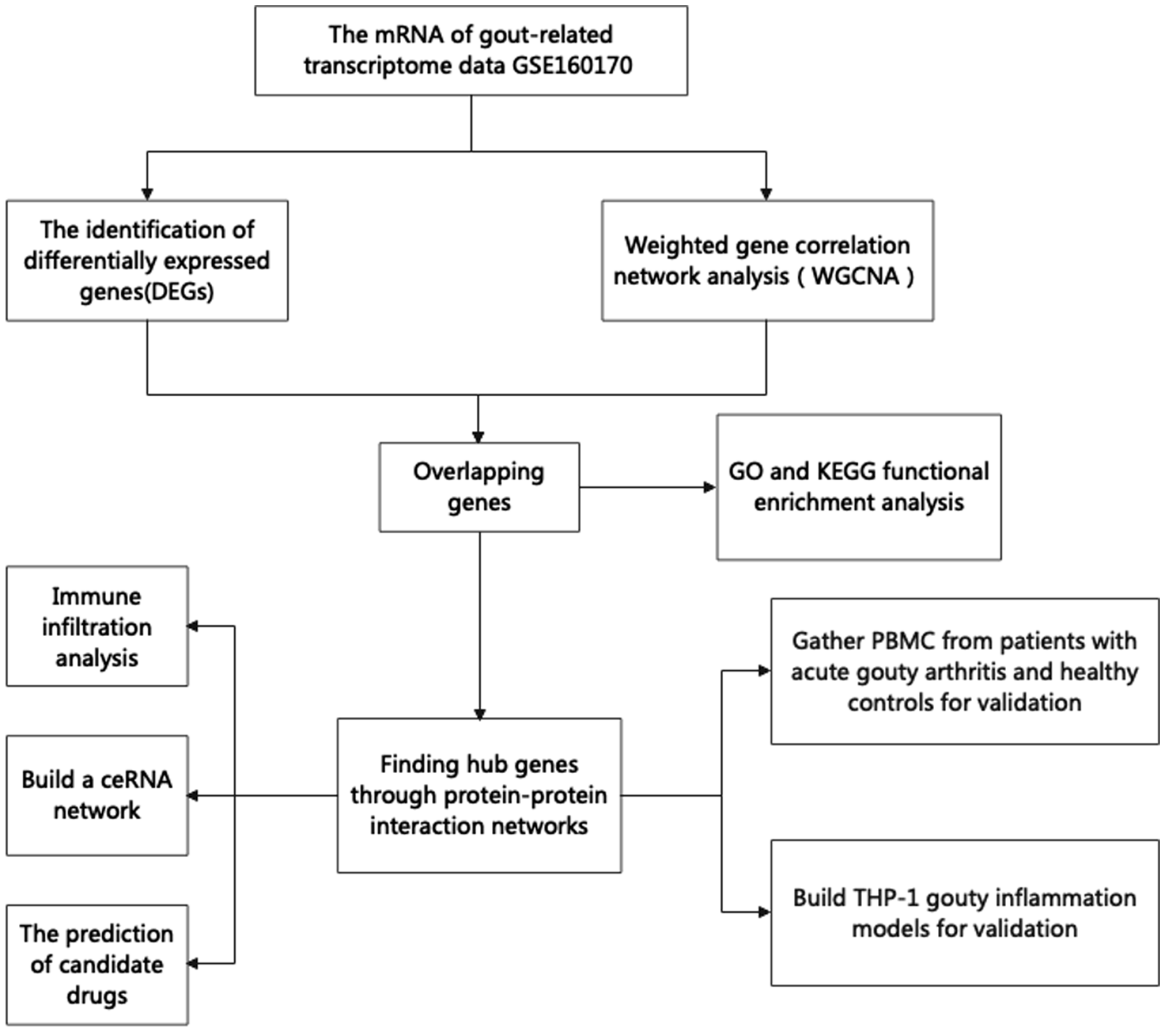
The flowchart of our research.

The NCBI-GEO database (https://www.ncbi.nlm.nih.gov/geo) is a free microarray/gene profile database. Gout-related noncoding RNAs, comprising differentially expressed lncRNAs and mRNAs, were obtained from the GEO database GSE160170. After screening the differentially expressed mRNAs using the “Limma” package in R 3.6.1, a P value< 0.05 and a |log2-fold change (FC)| >2 was used. The “ggplot2” package was used to visualize volcanic activity. The flowchart of our study design is shown in **Figure 1**.

### 2.2 Weighted gene correlation network analysis

The “WGCNA” package, a rigorous biological strategy that uncovers highly synergistic mRNAs by establishing scale-free networks and combining them with clinical data, was used to identify the most relevant gout genes.

### 2.3 Enrichment analysis

GO and KEGG functional enrichment analyses were subsequently used to investigate the biological activities and important pathways associated with the significant enrichment of DEGs. Based on the “clusterProfiler” package, functional enrichment analysis was carried out, and the “ggplot2” package was used to visualize the results. The standard was established at a p value<0.05.

### 2.4 Protein–protein interaction (PPI) network

The STRING database (http://string.embl.de/) is a biological database used to retrieve protein interactions. PPIs of DEGs were constructed with a confidence score of ≥0.7 based on the STRING web tool. Afterward, the Mcode and cytoHubba plugins in Cytoscape 3.5.1 software were used to screen and visualize the hub genes of the PPI network.

### 2.5 Immune cell infiltration analysis

The “GSVA” package was used to conduct ssGSEA on the GSE160170 dataset. Three files were used for GSVA: the transcriptome matrix, the immune cell type list, and the grouping data. Grouping box plots were created to show the differences in immune cells between the two groups. Heatmap was utilized to show the link between hub genes and immune cells.

### 2.6 Construction of the ceRNA network

The “MultiMir” package was used to search the MIRTARBASE database for potential upstream miRNAs. Then, miRNA−lncRNA data were downloaded from the STARBASE database (STARBASEV3_HG19_CLIP-SEQ_LNCRNA_ALL) with Pancancernum>10 and Clipexpnum>5, and Cytoscape 3.5.1 software was used to visualize the ceRNA network.

### 2.7 Patient samples

Twelve healthy control individuals and male patients with primary gout were recruited from Guangxi Medical University’s First Affiliated Hospital. Our research was approved by the First Affiliated Hospital of Guangxi Medical University’s Ethics Committee[2023-E710-01]. The gout diagnosis satisfied the 2015 American College of Rheumatology/European League Against Rheumatism gout classification criteria (16). We evaluated each patient by determining their sex, age, serum urate (SUA), white blood cell count (WBC), neutrophil granulocyte counts (NEU), lymphocyte counts (LYM), monocyte counts (MONO), total cholesterol (TC), triglycerides (TG), high density lipoprotein cholesterol (HDL-C), low density lipoprotein cholesterol (LDL-C) and blood glucose (GLU) (**Table 1**).

**Table 1:** The clinical data of gout patients.

	HC	GA	p
Sex (F/M)	0/12	0/12	
Age (year)	44 ± 5.8	45 ± 13	0.886
SUA (μmol/L)	391 [66]	519 [121]	0.001 **
WBC (×10^9^/L)	6.3 [1.8]	9 [5.9]	0.024 *
NEU (×10^9^/L)	3.2 [0.93]	6.1 [5.2]	0.002 **
LYM (×10^9^/L)	2.2 ± 0.46	1.6 ± 0.77	0.0347 *
MONO (×10^9^/L)	0.43 [0.17]	0.91 [0.36]	0.006 **
TC (mmol/L)	5.3 ± 0.82	5.9 ± 1.3	0.18
TG (mmol/L)	1.6 [0.87]	1.7 [1]	0.603
HDL-C (mmol/L)	1 [0.25]	1.1 [0.14]	0.603
LDL-C (mmol/L)	3.2 [1]	3.9 [0.81]	0.133
GLU (mmol/L)	4.9 [0.36]	5.4 [1.9]	0.453

* P<0.05, ** P<0.01.

### 2.8 THP-1 gout inflammatory model

THP-1 cells (Procell, Wuhan, China) were cultured in RPMI-1640 medium (Procell, Wuhan, China) supplemented with 10% FBS (Gibco, USA), 0.05 mM β-mercaptoethanol (Solarbio, Beijing, China), and 1% P/S (Solarbio, Beijing, China). THP-1 cells were stimulated for 24 hours to produce macrophages using 100 ng/L phenol 12-myristate 13-acetate (PMA; Sigma−Aldrich USA) (17–19).

PMA-treated THP-1 cells were stimulated with 0 µg/ml, 50 µg/ml, 100 µg/ml, or 200 µg/ml MSU crystals, and the optimal stimulation concentration was selected by CCK8 (NCM, Suzhou, China) to establish the gout inflammation model.


Cell viability (%) = [A(spiked)−A(blank)]/[A (0 spiked)−A(blank)] × 100

### 2.9 RT−qPCR

All primer sequences are shown in **Table 2**, and GAPDH was used as an internal reference. RNA was extracted from peripheral blood mononuclear cells (PBMCs) from human blood and THP-1 cells using TRIzol reagent (Takara, Japan), and cDNA was produced by reverse transcription using HiScript^@^III RT SuperMix for qPCR (+gDNA wiper) (Vazyme, Nanjing, China). The target genes were amplified and detected via Stepone Plus (Thermo Fisher Scientific, USA) using ChamQ Universal SYBR qPCR Master Mix (Vazyme, Nanjing, China), and the relative mRNA expression of the hub genes was calculated via the ^2−ΔΔCt^ method.

**Table 2:** The primer sequences of different genes.

gene name	sequence (5’-3’)
GAPDH-F	AATCAAGTGGGGCGATGCTG
GAPDH-R	GCAAATGAGCCCCAGCCTTC
CXCL8-F	ACTGAGAGTGATTGAGAGTGGAC
CXCL8-R	AACCCTCTGCACCCAGTTTTC
CXCL2-F	CTCAAGAACATCCAAAGTGTG
CXCL2-R	ATTCTTGAGTGTGGCTATGAC
CXCL1-F	GCCCAAACCGAAGTCATAGCC
CXCL1-R	ATCCGCCAGCCTCTATCACA
IL1β-F	GTTCCCTGCCCACAGACCT
IL1β-R	TGGACCAGACATCACCAAGC

### 2.10 Western blot

RIPA high-efficiency tissue cell lysates and 1% PMSF proteinase inhibitor (Solarbio, Beijing, China) were used to lyse the THP-1 cells. A bicinchoninic acid (BCA) kit (Beyotime, Shanghai, China) was used to measure the protein concentration. After being separated via 15% sodium dodecyl sulfate−polyacrylamide gel electrophoresis (SDS−PAGE), the proteins were transferred to a polyvinylidene difluoride (PVDF) membrane. The PVDF membranes were blocked with 5% skim milk containing Tris-buffered saline/Tween 20 (TBST) for 1 hour before being incubated at 4°C for 18 hours with antibodies against CXCL8 (1:800; Proteintech, Wuhan, China), CXCL2 (1:800; Bioss, China), and CXCL1 (1:800; Proteintech, Wuhan, China). The membrane was incubated with secondary antibody at room temperature for one hour (anti-rabbit immunoglobulin G, 1:12,000; Absin). Enhanced chemiluminescence (ECL) (Biosharp, China) was used in an imaging device (iBright FL1000, Thermo Fisher Scientific, USA) to visualize the bands.

### 2.11 ELISA

Following the manufacturer’s instructions, an ELISA kit (Solarbio, Beijing, China) was used to measure IL-1β secretion into the serum by THP-1 cells.

### 2.12 Prediction of potential drugs

With the use of the Enrichr platform (https://amp.pharm.mssm.edu/Enrichr/), we were able to access the Drug Signature Database (DSigDB), which contains a variety of publicly available drug-related gene expression data, and the candidate drugs we obtained were sorted from smallest to largest based on adjusted p values.

### 2.13 Statistical analysis

Ordinary one-way ANOVA was utilized for multiple group comparisons. When two groups were compared, the independent Student’s t test was used for normally distributed variables, and the Mann−Whitney U test was used for nonnormal variables. The median was used to represent nonnormal distributions, and the mean ± standard deviation was used to express data that approximated a normal distribution. SPSS26 was used for all the statistical tests, and P values less than 0.05 were regarded as statistically significant. *** P<0.001, ** P<0.01, * P<0.05, ns insignificance.

## 3 Results

### 3.1 Screening and identification of DEGs

**Figure 2:**
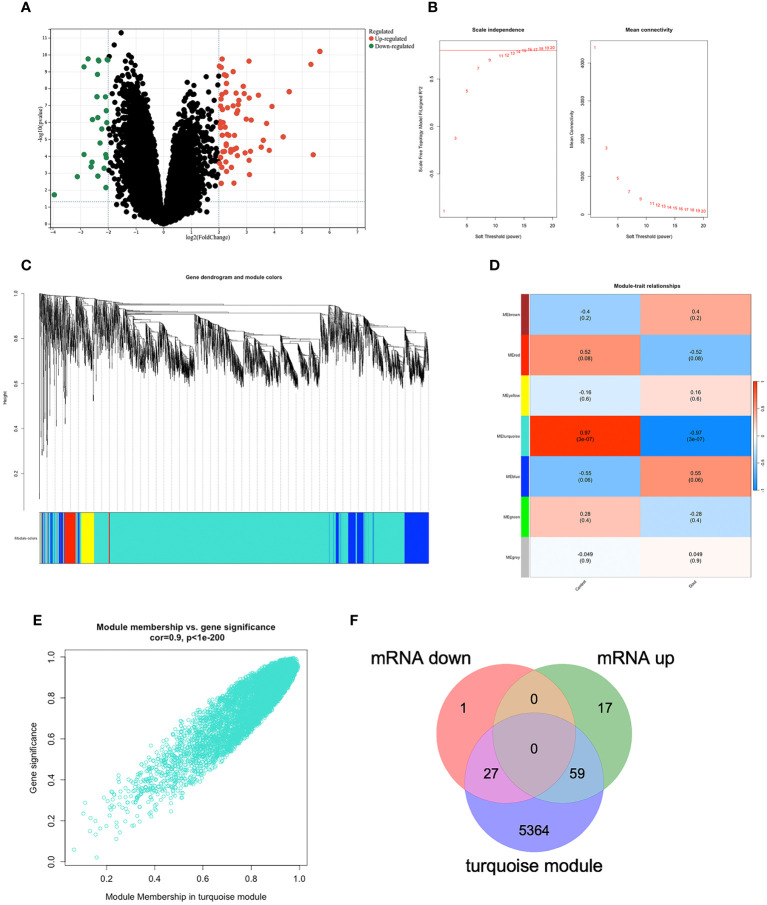
DEGs in GSE160170. **(A)**Volcano map of GSE160170, P<0.05 and |log fc|>2, with red indicating high expression, green indicating low expression, and black indicating no difference. **(B)** β=15 was selected to establish a scale-free network. **(C)** The correlations between gene module and clinical features, each module contains the corresponding correlation coefficient and p-value. Turquoise module has the highest correlation with gout. **(D)** A clustering dendrogram that illustrates how the genes are grouped within the module. **(E)** The correlation in turquoise module. **(F)** 27 mRNA were down-regulated, and 59 mRNA were up-regulated between DEGs and turquoise module.

We applied the only microarray dataset for human primary gout in the GEO dataset, GSE160170 (https://www.ncbi.nlm.nih.gov/geo/query/acc.cgi?acc=GSE160170), and the GPL21827 data platform (https://www.ncbi.nlm.nih.gov/geo/query/acc.cgi?acc=GPL21827), to screen the DEGs. We then analyzed the microarray data of mRNAs using the “Limma” package, visualizing DEGs (**Figure 2A**) with volcano plots based on P<0.05 and |log fc|>2. Each point on the plot represents a separate gene. Red represents upregulated genes, green represents downregulated genes, and black represents nondifferentially expressed genes. The GSE160170 dataset included 28 downregulated and 76 upregulated mRNAs.

### 3.2 Identifying the most relevant module

Using the WGCNA algorithm, we constructed a coexpression network and modules for the GSE160170 dataset. We set the soft threshold power β to 15 (R^2 = ^0.8) to construct a scale-free network (**Figure 2B**). Following that, a clustering dendrogram demonstrating the arrangement of the genes within the module (**Figure 2C**). After the acquisition of seven functional modules (**Figure 2D**), the turquoise module showed positive correlations between module membership and gene importance (**Figure 2E**) and was the most strongly connected and statistically significant module linked to primary gout (**Figure 2D**). Eighty-six hub genes were ultimately obtained by intersecting the turquoise module with the DEGs (**Figure 2F**).

### 3.3 GO and KEGG enrichment analyses of the hub genes

**Figure 3:**
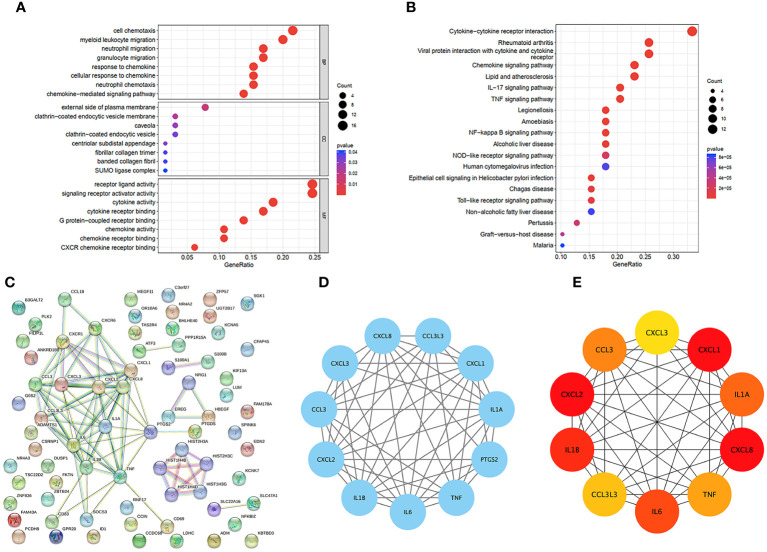
GO and KEGG functional enrichment analysis of DEGs in GSE160170. **(A)** Bubble diagram of GO enrichment in biological process terms, cellular component terms, and molecular function terms. **(B)** Bubble diagram of KEGG enriched terms. **(C)** The PPI network which contained 73 nodes and 86 edges, an average node degree of 2.36, an average local clustering coefficient of 0.379, and a PPI enrichment p-value of<1.0e-16, was visualized in STRING. **(D)** The densest connected region in the PPI network was identified using MCODE. **(E)** Top 10 genes with the highest degree values were found using CytoHubba_MCC and the depth of the color correspond to the weighted score.

We were able to explore the main pathways enriched by the hub genes using the “clusterProfiler” package. The DEGs were divided into three categories according to their GO annotations (**Figure 3A**): molecular function (MF), cellular composition (CC), and biological process (BP). The top 8 pathways were chosen for further evaluation based on the number of enriched genes and P value. For the biological process (BP) category, DEGs were associated mainly with cell chemotaxis. For the cellular component (CC) category, the main pathway was on the external side of the plasma membrane. For the molecular function (MF) category, DEGs were involved mainly in receptor ligand activity and signaling receptor activator activity. KEGG analysis (**Figure 3B**) revealed that the DEGs were associated mainly with cytokine−cytokine receptor interactions.

### 3.4 PPI network analysis of the hub genes

To identify important key genes, we entered 86 hub genes into the STRING database (**Figure 3C**). The minimum required interaction score was a high confidence interval of 0.700. Eventually, we obtained a PPI network with 73 nodes and 86 edges. The average node degree was 2.36, and the average local clustering coefficient was 0.379. The PPI concentration p value was less than 1.0e-16. The results were imported into Cytoscape for network visualization and analysis via the cytoHubba_MCC algorithm (**Figure 3E**). We chose the 5 most highly expressed genes—CXCL8, CXCL1, CXCL2, IL1β, and IL6—for the following study. We also performed a module analysis using the Mcode plugin (**Figure 3D**) and ultimately discovered that these 5 key genes were found in the most crucial modules.

### 3.5 Immune cell infiltration analysis

**Figure 4:**
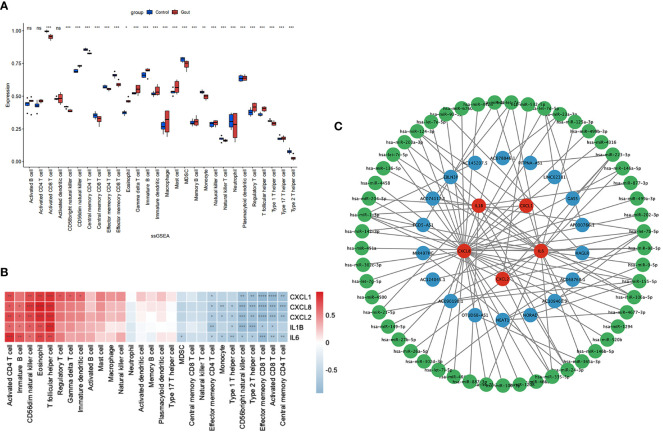
Immune infiltration analysis and CERNA networks. The distribution of 28 types of immune cells in GSE160170 are shown in the boxplot **(A)** and heatmap **(B)** show the relationship between hub genes and immune cells. **(C)** CERNA networks associated to hub genes have been built. Green denotes microRNA, blue denotes lncRNA, and red denotes mRNA. **** P < 0.0001, *** P < 0.001, ** P 0.01, * P < 0.05, ns insignificance.

We used the ssGSEA algorithm to assess the association between immune cell infiltration and patients with primary gout. The results of our study indicate that (**Figure 4A**) patients with primary gout exhibited greater levels of CD56dim natural killer cells, eosinophils, gamma delta T cells, immature B cells, immature dendritic cells, macrophages, mast cells, memory B cells, natural killer cells, plasmacytoid dendritic cells, regulatory T cells, T follicular helper cells and type 17 T helper cells; however, patients with primary gout also exhibited lower levels of activated CD8 T cells, CD56bright natural killer cells, central memory CD4 T cells, central memory CD8 T cells, effector memory CD4 T cells, effector memory CD8 T cells, MDSCs, monocytes, natural killer T cells, neutrophils, type 1 T helper cells, and type 2 T helper cells. We also found that (**Figure 4B**) these hub genes were positively correlated with immune cells such as activated CD4 T cells, immature B cells, CD56dim natural killer cells, Eosinophils and T follicular helper cells, and negatively correlated with immune cells such as effector memory CD4 T cells, monocytes, type 1 T helper cells, CD56bright natural killer cells, type 2 T helper cells, effector memory CD8 T cells, activated CD8 T cells and central memory CD4 T cells. These findings may also suggest a potential target for immunotherapy.

### 3.6 Construction of the ceRNA network

CeRNAs were found to be related to mRNAs, lncRNAs, and microRNAs, and we found 53 miRNAs and 18 lncRNAs that correlated with our hub genes (**Figure 4C**). Cytoscape was subsequently used to display the network.

### 3.7 Building a model of gouty inflammation

**Figure 5:**
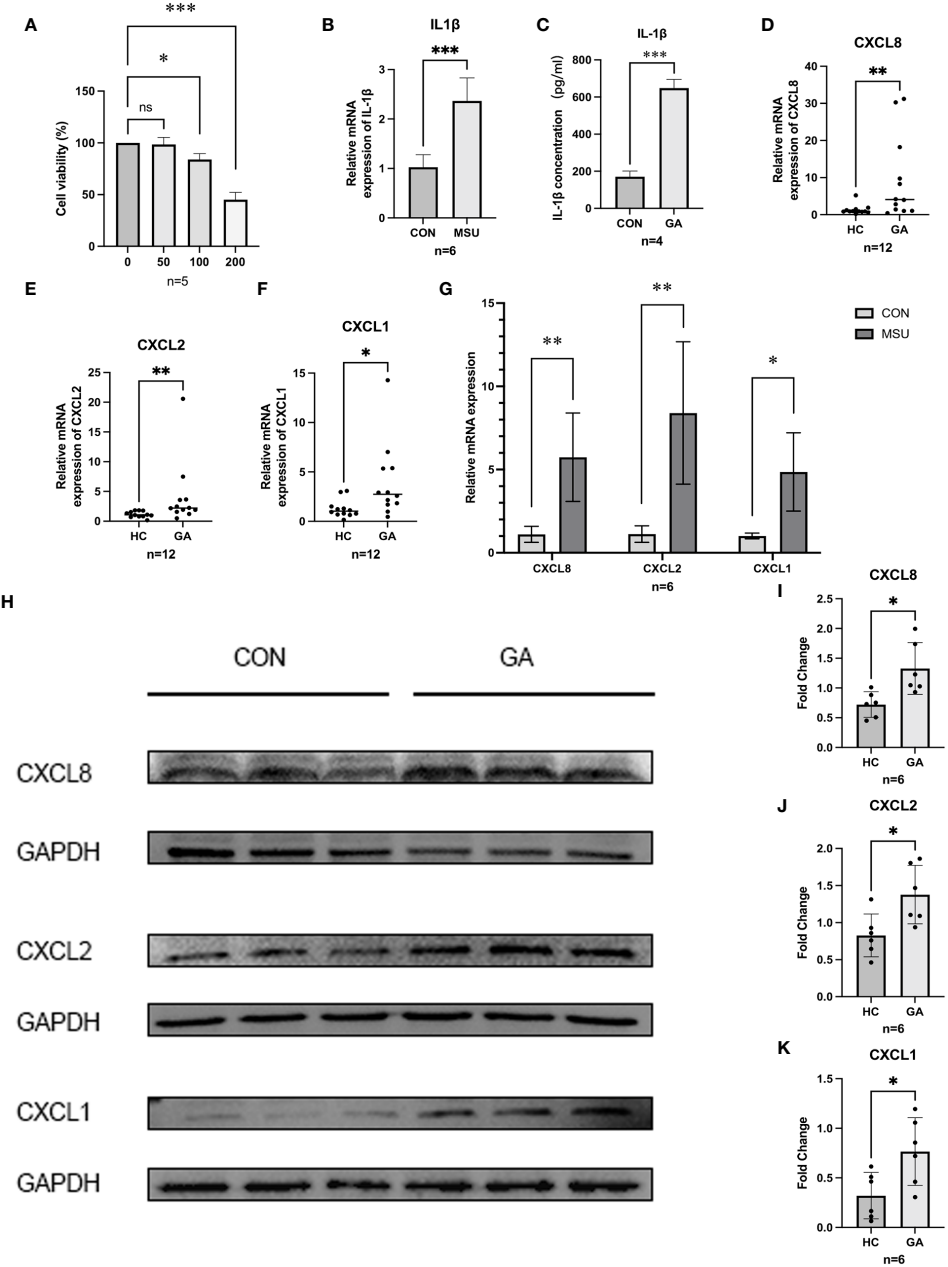
Gene expression and validation. **(A)** CCK8 was used to detect the proliferation of MSU on THP-1 cell activity. **(B,C)** ELISA and RT-qPCR were used to detect the release of IL-1β in THP-1 cells. **(D–F)** The relative mRNA expression of CXCL1, CXCL2, and CXCL8 in crowds. **(G)** The relative mRNA expression of hub genes in THP-1. **(H–K)** Protein level expression of hub genes. *** P < 0.001, ** P < 0.01, * P < 0.05, ns insignificance.

MSU crystals (0 µg/ml, 50 µg/ml, 100 µg/ml, and 200 µg/ml) were added after THP-1 cells were induced into macrophages. The CCK-8 assay results indicated that the viability of THP-1 cells decreased with increasing MSU concentration (**Figure 5A**), and 100 µg/ml MSU crystals were chosen for the generation of a gouty inflammation model in THP-1 cells. Compared with those in the group not treated with MSU crystals, RT–qPCR (p=0.0001) and ELISA (p<0.0001) showed that the expression of IL1β was significantly greater and that our gout inflammatory model was successfully established (**Figures 5B, C**).

### 3.8 The expression of hub genes in the population and in THP-1 cells

After PBMCs were harvested from five healthy individuals and six patients suffering from acute gouty arthritis, RT−qPCR revealed that patients with AGA expressed significantly more CXCL8 (p = 0.0173), CXCL2 (p = 0.0087), and CXCL1 (p = 0.0303) than healthy control individuals did (**Figures 5D–F**). After further validation of the expression of CXCL8, CXCL2, and CXCL1 in the THP-1 gouty inflammation model, MSU crystal stimulation was found to increase the expression of CXCL8, CXCL2, and CXCL1 compared to that in the group not stimulated with MSU crystals according to the RT−qPCR (**Figure 5G**) and WB (**Figures 5H−K**) results.

### 3.9 The prediction of candidate drugs

To anticipate potentially useful medications, we employed the Enrichr platform, which is based on the DSigDB database. The top ten candidate compounds were chosen based on their adjusted p values (**Table 3**).

**Table 3:** Drug prediction of the hub genes.

Term	Odds Ratio	Combined Score	P value	Adjusted P value
Profenamine PC3 UP	99640	2817407.04	5.25E-13	8.13E-10
CP-690334-01 PC3 DOWN	4994.75	131568.84	3.63E-12	2.81E-09
Muramyl Dipeptide CTD 00005307	4439.33	115109.77	5.48E-12	2.83E-09
Promethazine PC3 UP	2662	64109.21	3.47E-11	1.35E-08
CROTONALDEHYDE CTD 00000669	99080	2328114.32	6.24E-11	1.93E-08
Acetovanillone CTD 00002374	1995.5	45915.33	1.02E-10	2.63E-08
Pizotifen PC3 UP	1813.73	41080.14	1.46E-10	3.02E-08
PHENCYCLIDINE CTD 00005881	98870	2220526.04	1.76E-10	3.02E-08
Suloctidil PC3 UP	1662.25	37100.52	2.03E-10	3.02E-08
1-NITROPYRENE CTD 00001569	98830	2202286.26	2.10E-10	3.02E-08

## 4 Discussion

Gout is a prevalent kind of metabolic arthritis in which urate crystals accumulate in joints and nonjoint tissues. The consumption of seafood, red meats and shellfish, fructose, sugar-containing soft drinks, and alcoholic beverages, particularly beer, increases the risk of gout (1). Acute arthritis, particularly in the big toe joints, is the most common sign of gout. Patients may experience excruciating pain at night along with joint swelling, redness, and burning. Other joints, such as the knees and ankles, may also become affected as the illness progresses. The identification of effective therapies is crucial for managing and preventing gout. The transcriptome sequencing dataset GSE160170 was used for analysis, and we concentrated on mRNAs. By identifying genes with differential expression, building a WGCNA network to identify gout-related mRNAs, and inputting these overlapping mRNAs into the STRING database to construct a protein interaction network, the Mcode module analysis and the cytoHubba_MCC algorithm identified CXCL8, CXCL1, CXCL2, IL-1β, and IL6 as important biomarkers. Since IL-1β and IL6 have been linked to gout in numerous studies (20–23), our main focus was on the connection between CXCL8, CXCL1, and CXCL2 and acute gouty arthritis.

Chemokines are typically intimately linked to the migration and distribution of white blood cells within the immune system. Immune cells respond to infection or injury by releasing chemokines, which instruct other immune cells to migrate toward the site of infection or injury to carry out their defensive role. The majority of chemokines are currently categorized as inflammatory since they are essential for regulating leukocyte recruitment during the inflammatory response (24). CXCL8, also known as interleukin-8 (IL8), was the first chemokine discovered (25) and is secreted by numerous cells, including monocytes, endothelial cells, and macrophages, in response to suitable stimulation to trigger an endogenous or external inflammatory response (26). In a study by Anna Scanu et al. (27), synovial fluids were collected from a range of patients with untreated arthritis. The authors reported the highest expression of IL-1β, IL-8, and IL-6 in gouty synovial fluid. Additionally, other researchers have shown that the IL-8 rs4073 T allele is linked to a significantly greater risk of primary gouty arthritis. These findings imply that CXCL8 may be a unique biomarker for gout. Several cohort studies have shown that higher CXCL8 levels in gout patients increase the risk of CVD and type 2 diabetes (28, 29). As a result, CXCL8 might be a crucial target for the management and prevention of gout patients with diabetes mellitus or CVD. Studies on the role of CXCL2 in gout are rare, although CXCL2 has a chemotactic effect on neutrophils during MSU crystal-induced neutrophil migration (30). CXCL1 is an essential chemokine that can be activated by activating the NF-κB signaling pathway (31). Numerous researchers were able to alleviate gout by reducing the expression of CXCL1 (32–34). Kyle Jablonski et al. reported that (35) regular, moderate physical activity prevents acute inflammation in a gout model through the downregulation of circulating neutrophils by TLR2 and the inhibition of serum CXCL1. Ouratea spectabilis, especially ouratein D, acts as an antigout drug by reducing the migration of total inflammatory cells, monocytes and neutrophils and reducing the levels of IL-1β and CXCL1 in synovial tissue (36).

THP-1 cells are human monocytes that may be induced into macrophages by PMA. PMA-treated THP-1 cells are common cellular models for investigating the mechanism of gout because they replicate the release of inflammatory cytokines by MSU-stimulated macrophages *in vitro*. We observed that the expression of CXCL8, CXCL1, and CXCL2 increased after PMA-treated THP-1 cells were exposed to MSU. These findings are consistent with the findings in gout patients and healthy individuals, suggesting that CXCL8, CXCL1, and CXCL2 may be the keys to preventing and controlling gout. Furthermore, we discovered ten related therapeutic targets using the Enrichr platform, and a strong and statistically significant link between profenamine and hub genes was found. Profenamine, had sedative properties, is mainly used to treat Parkinson’s disease (37). At present, there are few studies related to Profenamine, numerous additional tests are still required to confirm the follow-up.

Although we used multiple methods to validate the data above, there are still some limitations. Our findings could lead to the development of new approaches for the treatment of gout; however, the precise regulatory mechanisms of CXCL8, CXCL1, and CXCL2 in gout remain unclear. Additionally, the sample sizes we obtained were too small, and the pharmacological therapeutic targets we identified have not yet been verified in animal models. Furthermore, we investigated the alterations in immune cells associated with gout. The ability of the ceRNA network to predict miRNAs and lncRNAs upstream of CXCL8, CXCL1, CXCL2, IL-1β, and IL6 may lead to the use of a novel diagnostic and prognostic marker for gout. In summary, CXCL8, CXCL1, and CXCL2 may be gout biomarkers, and this research may lead to novel approaches for the clinical management of individuals with gouty arthritis.

## Data availability statement

The datasets presented in this study can be found in online repositories. The names of the repository/repositories and accession number(s) can be found in the article/supplementary material.

## Ethics statement

The studies involving humans were approved by the First Affiliated Hospital of Guangxi Medical University’s Ethics Committee. The studies were conducted in accordance with the local legislation and institutional requirements. The ethics committee/institutional review board waived the requirement of written informed consent for participation from the participants or the participants’ legal guardians/next of kin because our samples were obtained in the past clinical diagnosis and treatment.

## Author contributions

XW: Conceptualization, Data curation, Methodology, Writing – original draft, Writing – review & editing. BY: Conceptualization, Investigation, Methodology, Project administration, Supervision, Writing – review & editing. TX: Data curation, Formal analysis, Investigation, Methodology, Writing – review & editing. YQ: Writing – review & editing, Data curation, Supervision, Formal analysis. YFQ: Conceptualization, Investigation, Methodology, Project administration, Resources, Supervision, Validation, Writing – review & editing. XL: Methodology, Project administration, Resources, Supervision, Validation, Visualization, Writing – review & editing. DL: Formal analysis, Methodology, Supervision, Writing – review & editing. XY: Funding acquisition, Methodology, Project administration, Resources, Supervision, Writing – review & editing.
